# What are practitioners' views of how digital health interventions may play a role in online child sexual abuse service delivery?

**DOI:** 10.3389/fdgth.2024.1325385

**Published:** 2024-03-20

**Authors:** Ethel Quayle, Matthias Schwannauer, Filippo Varese, Kim Cartwright, Will Hewins, Cindy Chan, Alice Newton, Prathiba Chitsabesan, Cathy Richards, Sandra Bucci

**Affiliations:** ^1^ School of Health in Social Science, University of Edinburgh, Edinburg, United Kingdom; ^2^ Division of Psychology and Mental Health, School of Health Sciences, Faculty of Biology, Medicine and Health, The University of Manchester, Manchester, United Kingdom; ^3^ Greater Manchester Mental Health NHS Foundation Trust, Manchester, United Kingdom; ^4^ NHS Lothian, Edinburgh, United Kingdom; ^5^ Pennine Care NHS Foundation Trust, Ashton-Under-Lyne, United Kingdom

**Keywords:** targeted cyberbullying, sexting, online grooming, cyber dating abuse, problematic internet use, nomophobia, the growth mindset intervention

## Abstract

**Introduction:**

Online child sexual abuse (OCSA) affects considerable numbers of children globally and is associated with a variety of mental health problems. Existing practitioner studies suggest that young people are infrequently asked about online abuse and practitioners have a fragmented understanding of the problems experienced or how they might approach them. There are very few evidence-based interventions that guide clinical assessment or practice. Digital Health Interventions (DHIs) have the potential to be an effective option where children and young people's services are challenged, including accessibility and anonymity. The aim of this study was to explore mental health practitioners' views of how DHIs may play a role in supporting young people who have experienced OCSA, and the role they can play in healthcare delivery.

**Method:**

In-depth qualitative interviews and one focus group were conducted with 25 child mental health professionals across two sites (Manchester and Edinburgh). Data was analyzed using reflexive thematic analysis.

**Results:**

Three overarching themes and 9 sub-themes were identified: (1) feeling a little bit lost; (2) seeing potential problems; and (3) knowing what works. Practitioners expressed interest in a DHI to support this client group and saw it as a way of managing waiting lists and complementing existing therapies. They felt that many young people would see this as a preferred medium to in-person therapy, would be empowering, and offers new ways of learning how to stay safe online. However, there were concerns about how much time would be needed by staff to deliver a DHI, anxieties about safety issues in relation to content and data protection, some of which may be unique to this population of young people, and concerns about the absence of a therapeutic relationship with vulnerable children.

**Discussion:**

Our findings indicated that practitioners were uncertain about working with children subjected to OCSA but were receptive to the possibility of using a DHI to support their practice and to reduce waiting lists. Concerns were expressed about the time needed for staff training and support as well as concerns over patient safety and the lack of evidence about the effectiveness of an unsupported DHI.

## Introduction

The Luxembourg Terminology Guidelines define both online sexual exploitation and sexual abuse as any form of sexual abuse of children which has a link to the online environment or is facilitated by internet communication technologies (ICTs) (1). More recently, the term “technology-assisted sexual violence” has been used in relation to adults (2–5) and “technology-assisted child sexual abuse” where children are involved (5–7). Such broad definitions mean that there is a lack of consensus about which activities comprise online sexual exploitation and sexual abuse and how they are measured. Online child sexual abuse (OCSA) is a significant problem in terms of its prevalence and impact (8), with a recent nationally representative study from the US with 2,639 young adults aged 18–28 indicating prevalence rates of 15.6%. Prevalence rates varied across difference presentations of OCSA; image-based sexual abuse, 11.0%; self-produced CSA images, 7.2%; non-consensual sexting, 7.2%; online grooming by adults, 5.4%; revenge pornography, 3.1%; sextortion, 3.5%; and online commercial sexual exploitation, 1.7%. Children and young people (CYP) aged 13–17 years were more likely to be targets across all groups (81.8% of the aggregate) with those who were 12 or younger seen in less than 16% of all categories. While the age of perpetrators was unknown for many of the respondents, it was noted that, where age was identified, those under 18 years comprised a large percentage, particularly in OCSA involving sexual images.

OCSA is associated with a variety of mental health problems (9–13), including Post-Traumatic Stress Disorder (PTSD) as well as behavioral problems (14, 15). However, for many CYP, in addition to mental health issues such as anxiety and depression, there appear to be emotional sequelae such as self-blame or criticism, along with an ongoing sense of loneliness, vulnerability and fears about other people becoming aware that, for example, sexual images were produced during the abuse (6, 16–19). This research challenges some of the stereotypes concerning OCSA and provides evidence that involvement of sexual images is likely to be associated with increased distress (20). Early publications identified that practitioners felt challenged when working with CYP who had experienced OCSA (21, 22) in part due to the limited evidence-based guidelines on how to respond to CYP presenting to services. Practitioners differed in how they conceptualized OCSA, how concerned they should be about these cases, and their understanding of the potential effects on the child (23). More recent studies from Canada acknowledged a lack of clinical guidance, with assessment and interventions influenced by routine practice along with anecdotal reports of cases of “conventional” child abuse in which the victim was photographed. These left practitioners unclear as to what questions to ask about OCSA along with a lack of confidence about the appropriateness of diagnostic assessments. As a result, clinicians rarely considered addressing these issues directly with CYP (24, 25). Across studies, there was agreement of a need for improvement in practitioners' understanding of how technology is used to exploit children, so that a more effective response to their respective client groups can be achieved (26, 27).

Further UK research has noted that child and adolescent mental health services (CAMHS) staff are not always aware of the most recent findings about the impact of OCSA or guidance available around digital safety (28, 29), with clinicians expressing awareness of, and concerns about, several digital risk issues, but also gaps in their knowledge and practice. Different factors played a role in whether they asked CYP about OCSA, which included lack of confidence in their knowledge and skills, a lack of resources which would facilitate engagement, and their motivation to change their routine ways of practicing. Another UK study explored how local services working with CYP (social care, health and the police) managed cases of OCSA (30). While there was an awareness of OCSA, it had a narrow focus with practitioners identifying types of abuse (e.g., online grooming) which led to clinicians not asking CYP questions about wider online risks or their antecedents. As with the UK CAMHS study (28), generic assessment tools were used, which tended to omit online risks unless specific safeguarding issues were identified. Furthermore, multi-agency collaboration was problematic as there was an absence of referral pathways and staff had few opportunities for specific training related to online risks. As with other studies, the emphasis for practitioners was on identifying and managing risk rather than understanding the experiences of CYP (31). However, there is some indication that this situation may be changing, with recent publications identifying which CYP should be asked about their online activities, when (and what) should be discussed, and with an understanding of possible outcomes; although, additional practitioner training is needed (32, 33).

There are few evidence-based interventions for CYP that target the sequelae of OCSA and there is very little training or resources for professionals to draw upon (34). There have been a number of digital health interventions (DHIs) to promote healthy romantic and sexual relationships with adolescents (35, 36), but fewer interventions for online sexual health (37) or adolescent sexual rights (38). A systematic review of education and awareness interventions to prevent OCSA indicated overall that there was some increase in safety knowledge but with limited impact on risky behavior (39). It has been noted that current online safety resources lack evidence of effectiveness and may include warnings that do not accurately reflect the dynamics of these sexual crimes (40). A teacher-led program of online activities (41) with Spanish schoolchildren was able to demonstrate changes across self-reported rates of online grooming and problematic Internet use. A further study (42) demonstrated that a “growth mindset intervention” was beneficial in promoting resilience for adolescents who had not experienced online victimization at pre-test, but it was not beneficial to those who had been victimized. A follow-up to this study used an online educational intervention (video and text and lasting less than one hour) which focused on online sexual grooming and compared it to a resilience-control intervention. For adolescents who had received the brief educational intervention, there was a reduction in sexual interaction behaviors (such as sharing or sending sexual photos or videos of themselves) with adults engaged in online grooming (43); this was not the case for the control intervention. While not without its limitations, the results of this study are encouraging, as they indicate that a brief DHI may be effective in not only increasing knowledge about one form of OCSA but also in reducing engagement with online perpetrators.

DHIs, used both with and without direct involvement by practitioners, have the potential to be an effective option where CYP's services are challenged and have advantages, including being accessible and anonymous, cost-effective, relevant to real-world contexts, and delivering high treatment fidelity (where the treatment in a research study is conducted consistently and reliably) (44). However, while the growth of DHIs has given an opportunity to address the increasing gap between demand and supply of CAMHS, they have yet to reach their full potential. A systematic review examined modes of delivery and facilitators and barriers for engaging CYP (45) and identified that CYP prefer DHIs with features such as videos and limited amounts of text, the ability to personalize content and connect with others, and demonstrated a high average retention rate of 79% (defined as the percentage of participants completing outcome measures for at least one follow-up time point). However, other reviews have indicated that adherence and engagement rates tended to be low in many studies, particularly those where interventions were completed in a person's own time, suggesting that DHIs are most likely to be useful for people already receiving mental health support (46).

Research examining the views and concerns of practitioners about DHIs is also important as previous research has suggested that practitioners may be the gatekeepers to promoting and supporting DHIs (47). A qualitative study with 48 mental health staff who work with people with experience of psychosis examined views about the use of digital tools for people accessing specialist services (48). The study concluded that to maximize their uptake, DHIs need to be uncomplicated and bring efficiencies in clinical workflows, with organizational support another key factor for their adoption.

Earlier studies had suggested a resistance by mental health practitioners to using technology-enabled approaches to support CYP because of issues related to the perceived absence of a personal connection between therapist and CYP (49, 50). Healthcare practitioners view digital tools as following, co-occurring with, or culminating in, other treatment approaches, in particular in-person therapies, rather than as stand-alone alternatives (51). While practitioners increasingly see themselves as adopters of digital health technology and see their value in healthcare delivery, factors such as lack of time to invest in their use, attitudes of some colleagues, data security concerns and lack of evidence about the effectiveness of digital tools limit their adoption (52–55).

### Aim of the current study

We explored mental health practitioners’ views of how DHIs may play a role in supporting CYP who have experienced OCSA, and the role they can play in healthcare delivery.

## Materials and methods

### Participants

Participants (*N* = 25) were recruited by posters and digitally across two UK sites (Manchester, Edinburgh) with National Health Service (NHS) CAMHS, a Sexual Assault Referral Service and a national e-therapy provider. Inclusion criteria were healthcare professionals currently working in the recruitment sites with a good understanding of the English language. The sample size was determined by paying attention to the study aims and in-depth exploration of a sample that has shared characteristics (56, 57).

### Design

A qualitative study design (thematic analysis) was used. Individual interviews (Manchester, UK, Edinburgh, UK) and a focus group (Edinburgh, UK) provided a range of accounts across healthcare practitioners. These were analyzed using reflexive thematic analysis [RTA (58, 59)]. RTA is a theoretically flexible interpretive approach to qualitative data analysis that facilitates the identification of patterns and themes within the data, but where the researcher plays an active role in knowledge production. Coding was examined by, and discussed with, members of the team (60).

The interview and focus group questions addressed beliefs about the strengths and challenges of delivering a DHI with our target group, expectations about what a DHI with our target group should look like, and how it can be integrated into current clinical practice. Practitioners' understanding of OCSA, its assessment and management, were also included but are not addressed within this publication. The interviews were semi-structured, and questions were open-ended with sequencing dictated by the flow of the exchange. Probes were used to aid further elaboration of responses. In the development of the study we consulted the consolidated criteria for the reporting of qualitative research (COREQ) with regard to reflexivity and study design (61).

### Procedure

The relevant Institutional ethics approval was granted (REC Number 21/WS/0160) and the protocol was registered at ClinicalTrials.gov (ClinicalTrials.gov Identifier: NCT05006053). Participants were approached through service managers of the relevant UK NHS Trusts and an e-therapy provider platform to ask for permission for an advert/flyer to be circulated to staff via email, their website, social media, newsletters, weekly bulletins, and announcements. Researchers also attended departmental meetings where interested staff were encouraged to email the researchers for a Participant Information Sheet and consent form. In addition, an advert was circulated via our research group's website and social media account. People who confirmed that they would like to take part in the study were given the choice to meet online individually or as part of a focus group. Consent was obtained verbally using an oral consent script. Interviews lasted approximately one hour and two hours for the focus group and were transcribed, anonymized and given an identifying code (also used to identify where extracts came from within the Results) and stored securely. Field notes and reflexive logs were kept throughout. Participants completed a brief demographic form once consent had been given and prior to the interview or focus group. No financial compensation was offered to participants. Data was collected between July 2021 and January 2022.

### Data analysis

Interviews were audio-recorded and then transcribed. Analysis was supported by the end-to-end encrypted software application Dedoose for qualitative and mixed methods research (62). A predominantly inductive approach was adopted. Data was open-coded, and meanings based on the interpretations made by respondents was emphasized. However, the questions asked in the interview and focus group, although used flexibly, meant that deductive analysis was also employed to ensure that the open coding allowed for the identification of themes that were meaningful to the research questions posed. Therefore, both semantic and latent codes were used and we followed the proposed recursive and iterative six-stage analytical process to facilitate coding and theme-identification: (i) familiarization with the data; (ii) generating initial codes; (iii) generating themes; (iv) reviewing potential themes; (v) defining and naming themes, and (vi) producing the report (56). The codes were primarily developed by EQ and SB working alongside each other and were further sense checked by WH and reviewed by members of the research team as the codes were developed. We coded according to the guidelines outlined for “reflexive thematic analysis” where coding is open without the use of any coding framework. Second coding was therefore not used and there was no attempt to determine inter-rater reliability (56). Instead, quality assurance of the coding, theme development and the final write up were guided by a recent tool for evaluating research quality (59).

## Results

Twenty five professionals were recruited across two UK sites (Manchester (*N* = 15) and Edinburgh (*N* = 10)). For staffing reasons, six members from the Edinburgh sample opted to be part of a focus group. A summary of participant characteristics is presented in Table 1. Sex was determined by asking participants to select one of the following categories: Male, Female, Non-binary; Transsexual and Other. The two sites were demographically similar.

**Table 1:** Summary of participant characteristics.

Overall sample	*N* (%)
Sex	
Female	22 (88)
Male	03 (12)
Ethnicity	
White British	19 (76)
White (any other background)	04 (16)
Asian	1 (04)
European	1 (04)
Area of Service	
CAMHS	15 (60)
Adolescent In-patient	04 (16)
Community	01 (04)
SARC	02 (08)
E-therapy	02 (08)

**Figure 1:**
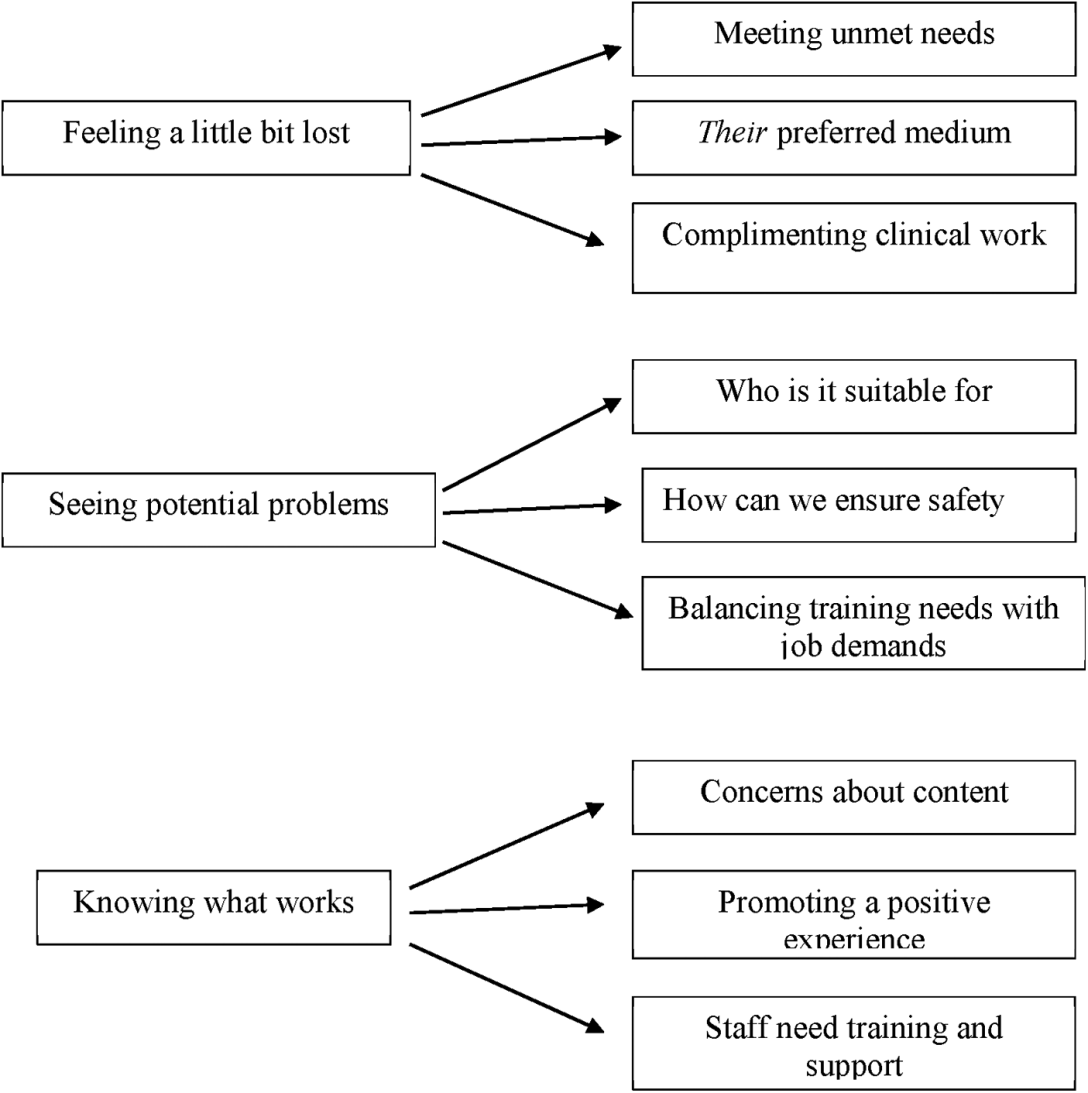
Visual depiction of themes and sub-themes.

Three overarching themes and 9 sub themes related to the research questions were identified: (1) Feeling a little bit lost; (2) Seeing potential problems; and (3) Knowing what works (Figure 1).

### Theme 1: feeling a little bit lost

The interviews and focus group explored practitioners' perceptions of how a digital intervention for CYP who had experienced online sexual abuse may be used in clinical practice. While all respondents demonstrated an awareness of OCSA and when identified the potential need for therapeutic intervention, they were less confident about how they would respond to a disclosure or referral, or what resources may be available for them to use in therapy. This is reflected in the following subthemes: Meeting unmet needs; complimenting clinical work and feeling confident about its use as psychoeducation.

#### Subtheme 1: meeting unmet needs

Given the time that the study data was collected (at the end of the pandemic), there was a lot of general discussion about remote interventions through video calls and apps, and the implications that might have for both CYP and practitioners. Practitioners drew on what they knew, and they discussed how existing DHIs were used when waiting lists were long and when in-person services were not readily available. The DHIs that they referred to largely targeted specific known clinical problems such as depression or anxiety and were often seen to be congruent with existing clinical interventions used by these practitioners. In this respect DHIs were mainly seen in a positive light although ambivalence was expressed about how they were really used by CYP. However, it was noted that unlike in-person therapy, DHIs were available to CYP when they needed them most, and this was felt to be particularly salient where OCSA had taken place:

*“As an app intervention it’s really readily available, something they can access at any time, easily download, and that there’s no judgement, they’re getting all of that information without having to go to like a teacher or a parent or, erm, a sexual health clinic, or something like that, so definitely I think that’s a huge advantage of it” (MAN-011)*.

This emphasized not only the importance of easy access but also the need for a non-judgmental stance towards CYP when seeking support for the disclosure of sensitive information. The focus by practitioners on the need for information (as opposed to intervention) was a recurrent topic throughout the interviews. The idea of a DHI that targeted OCSA appeared to be of particular interest to practitioners given that they felt that they had few resources when responding to some of the CYP they were working with:

*“Erm, and it would be good to have to know about that and have that, have that as an option, as a tool, toolkit, erm, but, erm, I suppose if, if in some ways that can be as accessible as it’s possible to be, I think that’s really, that’s really good, bearing in mind this group of young people that we, that we’re involved with, who are, notoriously difficult to meet with” (MAN-003)*.

The focus often moved between meeting the needs of CYP but also the needs of practitioners in either providing an option to in-person therapy or providing them with the appropriate tools for working with CYP who may otherwise be reluctant to seek help or turn up for appointments. This was also echoed with reference to DHIs reducing the time spent on waiting lists and potentially meaning that no further appointments would be needed. In the following extract it appears that DHIs also opened the possibility of CYP talking to their parents about experiences of OCSA, therefore reducing or supplementing the need for a therapist:

*“I think that that could really help with people waiting to be seen, which might mean that by the time they reach the top of the waitlist they might not need to be seen, or at, at least it might have opened up lots of conversation between them and parents” (ED-09)*.

In this sense, an OCSA DHI seemed to be meeting multiple needs for practitioners who had few resources to draw upon, as well as meeting CYP's needs for information sourced when needed and in privacy. What was not discussed, even though respondents talked about potential harms associated with OCSA, was the therapeutic goals of an DHI, outside of providing information, and no reference was made to the needs of these CYP being like those of children who had been sexually abused or exploited in the offline environment.

#### Subtheme 2: *their* preferred medium

Alongside the practical advantages of DHIs, there was also discussion about this being a preferred therapeutic medium for many CYP in part because it was more engaging than traditional talking therapies and, in the context of OCSA, avoided the intense embarrassment of having to discuss the topic:

*“I think your advantage there is that it is exactly the medium that young people are using, erm, so you’re gonna get interaction, so you’re gonna get more interactions. Erm, it means as well that young people can, can get this information, there’s help with that, having to go through that excruciatingly embarrassing face to face contact with someone” (MAN-013)*.

While in this sense the focus was very much on meeting the young person's needs, rather than the needs of practitioners, this may also reflect a level of discomfort for practitioners as well in terms of how they might respond to a disclosure. Many of the practitioners expressed concerns that they really did not understand how CYP engaged with technology “*I mean, I only know about TikTok because I worked at a school and the kids were talking about it, I never knew that this existed* “*(MAN-007)* and highlighted what was felt as a digital divide between practitioners and the CYP they worked with. However, the feeling that this was *their* preferred medium did open up benefits that might be seen as missing, or more difficult to ensure, through in-patient meetings. Access, privacy and increasing the likelihood of engagement were frequently referred to:

*“Why isn’t there an app for it? You know, erm, so yeah I think generally there’s been a sort of a language sense it’s, it’s what they’re used to… you know as in like opening an app and, and being busy, it’s not gonna draw attention, as it were… I think things that can be done privately, I think that’s always a, a bonus, a benefit” (MAN-008)*.

Whilst participants felt some concerns about a DHI not providing the same therapeutic support if delivered by in-person interventions for CYP who had experienced OCSA, there was also consideration that a DHI that they could access in their own time and chosen environment might not only provide a space and time to engage but promote feelings of safety without the anxieties of close, physical proximity with a clinician:

*“I think also for young people… perhaps the kids who’ve, who’ve been abused,.. who have that hypervigilance around them, they, they could perhaps find the physical proximity of an adult quite intimidating, there might be something about the digital forum that actually makes them feel a little bit safer because the person is, is on a screen rather than physically in the room” (MAN-001)*.

The acknowledgement of OCSA as sexual abuse, but the assumption of proximity to people being problematic, is noteworthy, given the number of cases presenting to these services which involved contact sexual abuse that would necessarily require in-person meetings. Paradoxically, not only were DHIs seen as a preferred medium for CYP, but also as an ideal space for teaching CYP who had experienced difficulties online how to manage their online engagement more safely:

*“I think increasingly our kids live in an online world don’t they, and we have to kind of adapt, they, they, they are very proficient online, they, they there are pitfalls of being proficient online and that’s exactly why we’re looking at things like online grooming, but actually probably, I guess some of the best ways to teach someone to be safe online is online” (MAN-010)*.

In this extract practitioners are positioned as people who need to adapt to the fact that CYP's lives are interwoven with technology and that this brings with it risks. It also may suggest that at present children's services are at the stage of “looking at” responses to OCSA, rather than “engagement with”. This is in marked contrast to some of the concerns expressed in relation to safety issues.

#### Subtheme 3: complimenting clinical work

For many of the participants, a DHI that had been developed for CYP who had experienced OCSA was seen as potentially complimenting the work that they were doing therapeutically rather than being a substitute for it:

*“… a kind of more like multimedia approach, definitely helps with engagement, and I think a lot of young people have really liked watching kind of psychoeducation videos rather than me just talking at them, because it feels a bit teachery… erm, and that might be integrated into existing therapy and then using apps alongside existing therapies for tools” (ED-010)*.

However, it was unclear how this integration would take place and what the therapeutic focus might be, although it was felt that a DHI offered a resource that would help CYP understand their experiences and learn to manage some of the difficult emotions associated with them even prior to therapy being available, “*… why is the re-experiencing happening, flashbacks, nightmares, avoidance…” (ED-007)*.

As noted, there was a lot of discussion about the DHI providing psychoeducation for CYP in the form of useful information and links to other services available to them, as well as education about how to navigate online information. Again, the focus was on information provision and education as opposed to therapy although this form of psychoeducation was seen as similar to what many practitioners tried to achieve in their own routine work with CYP:

*“… in terms of the psychoeducation bit, I think that’s vitally important. So some of those lessons… take home messages that we always try to put in for young people about trying to protect those boundaries… (MAN-009)*.

Practitioners also discussed benefits for themselves when navigating how to support those who have experienced OCSA, which again suggested at times feelings of professional uncertainty when confronted with these cases:

*“ I’m not sure I would feel like it’s extra work, personally, I think I would, I know sometimes I’ve felt a little bit lost, and, and trying to plan what I do with a young person specifically intervention wise, probably not now, but, historically I’ve had times where I’ve sort of gone, I don’t really, I’m, I’m stumped now, I’m not quite sure what to do, we’re having the same one to one sessions every single week, whereas actually what this could offer is a really targeted intervention from both sides.” (MAN-10)*.

For some practitioners using a DHI was envisaged as something akin to working with a co-therapist where the DHI was used as a form of “two-pronged therapy” (*(MAN-016)* to supplement and focus more routine clinical practices.

### Theme 2: seeing potential problems

There was a lot of discussion of concerns about using DHIs, both in general and more specifically in relation to OCSA. Much of this related to a perceived loss of control about how CYP may engage with the DHI, or the device, and is seen in the following sub-themes: Who is it suitable for; How can we ensure safety, and Balancing training needs with job demands.

#### Subtheme 1: who is it suitable for?

Practitioners drew on their experiences of how digital platforms had been used during the pandemic and were alert to the clinical limitations of online settings and therapeutic DHIs. Some of these limitations related to inequalities of access to both devices and data, particularly where services were based in areas of high social deprivation. This raised questions of how a DHI could be used when access was not guaranteed, or where there was limited physical space to allow for privacy. The latter may be particularly relevant where CYP had not disclosed to care givers that OCSA had taken place and wished to keep this information private:

*“I think, one thing we already look at, is really important around any app or electronic intervention is, is, is, er, poverty and to do with data poverty or equipment poverty as well. So a lot of young people, their families are using data, or have poor wifi links or poor equipment, so that, that’s one to look at. And the other thing as well is, it’s private space, so a lot of the families we work with, they don’t have a private space to be able to do this” (MAN-012)*.

However, practitioners also discussed the specific needs of children exposed to OCSA when considering using or recommending a DHI. The suitability of a DHI where abuse had taken place online raised multiple issues for practitioners that went beyond a simple consideration of the DHI content itself:

*“… a few pictures circulated around social media and she’d actually deleted all of her social media accounts and she also won’t pick up the phone… maybe there are practical barriers of people’s relationship with the digital world and technology and apps might be distressing or unpleasant in it, in itself, if that’s also what, where the, the domain in which they experienced the abuse” (ED-FG1-006)*.

The issue of suitability was touched upon by a number of practitioners, particularly as it meant that CYP were being directed to go online rather than diverting them to what were considered healthier or more pro-social activities. While “going online” might have been seen by many practitioners to be the preferred option for CYP, this was certainly not the case for practitioners themselves. Being online was seen as the problem and therefore staying offline may also be the solution, or at least part of the solution:

*“Erm, the disadvantages are obviously, that, you know, it’s online sexual abuse… the problem was them being online and then we’re saying, ah, how about you go online and use this app, when actually, you know, it will be better to be like, you know, life doesn’t, life isn’t online, let’s try bring you out of that, let’s do more activities with you, let’s do, things in person rather than online, and then obviously be like, but use this online app” (MAN-015)*.

Questions were also raised about the suitability of a DHI for CYP with intellectual disabilities or Autism Spectrum Disorder (ASD), and whether younger children would be able to process and respond to the information provided by the DHI. To some extent this related to the developmental stage of the child or their ability to process information and raised some interesting issues overall about how CYP with disabilities may be marginalized in their ability to use any form of digital communication:

*“I mean for instance, I have a case of a young person that, erm, they have Autism, and so kind of communicating online can be really tricky, er, ‘cos there’s lots of cues that they already don’t pay quite attention, but then it is even less cues online” (MAN-002)*.

However, most practitioners thought that while there may be challenges, for many CYP with, for example, ASD, using a DHI may be in fact be seen more favorably than in-person therapy because it may remove some of the difficulties that come from social interactions. However, this was talked about in terms of general DHI usage rather than specifically in relation to CYP who have been subjected to OCSA.

The way that online behavior changes interpersonal relationships was seen to be a core element of online sexual abuse and that, “*it's really important to redress that balance” (ED-GP1-006)* through experiencing a positive and nurturing relationship with a clinician*.* The concern about the “missing practitioner” was seen to be critical in relation to managing content (as well as the context) of material that might trigger painful memories and be associated with high levels of distress in the young person:

*“there’d have to be therapeutic input or some involvement as well because if it was just a stand-alone and they were going through an online app that then is bound to be triggering, it’s gonna be difficult, it’s almost like, well who do I touch base with and who, who am I connected to that kind of makes me feel safe, as opposed to, right I’m just working through this and I’m doing it quite, you know” (MAN-009)*.

This concern for many practitioners related to the absence of a therapeutic relationship when using an unsupported DHI and how this may in fact place a young person at risk of exposure to content that would cause further harm. It was also felt that this potentially undermines what might otherwise be important therapeutic goals where OCSA had taken place concerning trust and the need to understand who can be trusted:

*“I guess, that is really kind of… it might affect someone’s trust in other people and affect kind of their other relationships, so, it might be trying, it might help them to kind of recalibrate who they can trust and, and, and notice you know what other people’s intentions are, because they might be skewed after experiencing something like that” (ED-010)*.

#### Subtheme 2: how can we ensure safety

This theme is clearly related to questions around the suitability of a DHI for CYP exposed to OCSA but there were also concerns about very practical safeguarding issues regarding access to a smartphone given that some CYP would have had their device taken off them as a response to the discovery of OCSA. This may have been related to police involvement or the decision by a parent or therapist:

*“… but with the police, a lot of times, especially when it’s online abuse, they will take their phones, and… so, unless parents can afford to buy young people a new phone, a lot of my clients, I can’t speak to because they don’t have a phone” (MAN-015)*.

Safeguarding issues also related to whether the DHI and its content was safe to use, or whether the content itself may “push” CYP to explore sexual content or engage in behavior that would put them at further risk. For example, using some of the functions on their smartphone to engage in sexual chat with someone, access pornography or share sexual photos. This seemed a particular issue when there were concerns about whether a young person may still be in contact with a perpetrator or sharing problematic sexual content with peers. The inability to easily control access to digital content and contacts, alongside how these relationships may be perceived differently by practitioners, parents and CYP raised very legitimate issues around the confidence that practitioners may have in the DHI and how it (or the device) may be abused:

*‘I would worry about potentially their risks, erm, and how that can be managed if they’re actively still seeing and involved with people who are perpetrators of abuse’ (MAN-010)*.

This issue clearly goes beyond the use of a DHI and increasingly is seen as a problem in the management of digital contact by a known adult where offline sexual abuse has taken place. Practitioners discussed whether they (and others) would have access to what CYP using a DHI may be doing, both in relation to DHI-usage itself but also online activity through other online applications. In this context the benefits of providing a private space for CYP through a DHI had to be considered in relation to the rights of these CYP and their carers to protection from online harms.

#### Subtheme 3: balancing training needs with job demands

While many practitioners felt that they lacked the skills or resources to work with CYP who had experienced OCSA, promoting a DHI, or using it within an existing therapeutic relationship or service, was seen to be potentially a source of additional work. This prompted a cost benefit analysis of whether it was worth the time and effort to learn about how a new DHI could be used therapeutically:

*‘Erm, I guess something that’s always tricky with apps and stuff is sustainability, erm, so if, you know, if I was a clinician and someone said, we’ve got this new app, do you want to learn about it, in the back of me mind I’d be thinking, I could, you know, is it worth, it is worth potentially learning about it, also, is it still going to be around in 12 months, ‘cos if it’s not, I can’t, I’m not gonna make time for learning that, because, it'll be something else in another 12 months” (MAN-006)*.

This probably reflects the proliferation of digital health interventions for CYP and a lack of information about the evidence for what works alongside the longevity of its availability to both practitioners and CYP. Some of this caution was not because it was considered unimportant, but rather that it meant taking onboard something new and, for people who felt technically ill-equipped, somewhat threatening. This was not discussed with specific reference to DHIs but rather to digital platforms and applications that they felt unfamiliar with. Anxieties about having the technical skills to manage the DHI were also evident across respondents. The implications of this was the need for additional training and how this would be managed by the organization and also the individual. Acknowledging this need was set alongside staff having to give up clinical time to training sessions and the realities of insufficient practitioners within services and rapid staff turnover meaning that training had to be ongoing:

*“I probably need some training into even just getting the app on, ‘cos at the moment my IT, they have been tested to the limit through the pandemic, so I would probably just even need the basics of how to access it (laughs) you know that kind of thing, so I, I definitely need that” (ED-007)*.

*“I think realistically we’re not, you’re not gonna get a lot of staff giving up time for a training day… particularly at the moment we’ve had a whole load of new staff so you could train and then, everyone moves on’ (ED-08)*.

What this also opened was discussion about how practitioners had a limited understanding of what OCSA is and how it is assessed and managed. The implications of this are important as participants felt unsure of the nature of the problem they had to deal with which left them feeling unskilled as to how to approach the topic with a young person let alone how they might suggest the use of a new DHI:

*“there is still, for a lot of people, practitioner’s included, uncertainty about what are terming kind of sexual abuse, online sexual abuse, you know, where are those thresholds, erm, where, where would we think someone would benefit from it, er, and I think some practitioners feel uncertain about how directly should I talk about this, how much should I ask, erm, so I think almost that sort of, you know, bit of encouragement, or reassurance about, how might we introduce this app” (MAN-008)*.

### Theme 3: knowing what works

Most of the practitioners interviewed had quite extensive experience of using technology to deliver therapy or DHIs as a substitute for, or in addition to, clinical sessions. This meant that they were able to reflect on how the DHI might be developed and service needs in terms of its use. This theme is made up of three subthemes: Concerns about the content; Promoting a positive experience, and Staff need training and support.

#### Subtheme 1: concerns about the content

The perceived need for some therapeutic support in relation to using a DHI developed for CYP who had experienced OCSA led to further concerns about how the content may be problematic and the need to ensure that there was transparency about this prior to exposing CYP to it. Unlike other DHIs this included anxieties about exposing CYP to content that would increase the likelihood of engagement in more risky online behavior, through searching or contact with others:

*“…given that we’re talking about sort of 14–18-year-olds, to what extent can they be as fully informed as possible, as to what the content would be. So I think it’s that, I don’t know whether there’s some slightly uneasy thing about, it’s this app that’s kind of gonna lead you into what more and more explicit material” (ED-001)*.

Concerns about the content of the DHI also related to the potential range, intensity and longevity of the OCSA experienced by individual CYP, which inevitably the DHI could not respond to. All DHI users would be exposed to the same content regardless of their age, sex, or severity of their abusive experiences. For some practitioners, these concerns were evidenced in their perceived need to be able to monitor the progress made by the young person they were working with and provide feedback based on how they were responding to, and working through, the module. The need for someone other than the young person to understand what they were doing in relation to the DHI was also framed in terms of offering support:

*“I’d like to see more than just the app, or if it is an app, that there’s a, I don’t know, parent/carer page on the app or something that, that they can download that helps them to, not monitor what the young person’s learning and doing, but, just clue them up a little bit, support them to support the kid” (MAN-016)*.

This was also addressed in concerns about privacy, particularly with regard to data protection and CYP having control over how their engagement with a DHI might be used:

*“I think it goes back to that transparency thing of like, you know, who are you gonna tell that I’ve engaged in this, kind of, are you gonna tell school, are you gonna tell GP, are you gonna tell parents, erm, what happens to what I do on it, erm, given all, you know, the focus on online kind of safety, I guess, how safe is this sort of thing, erm, how do they know that it’s a, a safe space” (MAN-008)*.

#### Subtheme 2: promoting a positive experience

A number of practitioners questioned how DHI developers could ensure adequate uptake of the DHI, and whether, for some CYP, the offer of a DHI may be seen as inferior to in-person therapy:

*“Erm, I think, I think that goes hand in hand to how we make families and young people feel listened to. If you just, if you kind of are listening to just respond to a query, or to a request or potentially somebody that’s quite angry because they have been on a waiting list for a long, er, and you don’t really listen, and you don’t really, really make that young person understand or they feel listened to, it, it’s more likely that they will feel you’re just fobbing them off to this online thing” (MAN-002)*.

This again was discussed in relation practitioners' experiences of using DHIs in relation to mental health difficulties and how this would impact on their willingness to refer CYP to use the DHI. These experiences, sometime negative, related to insufficient staff time, resources and support to familiarize themselves with new DHIs and fears that a DHI for CYP exposed to OCSA was yet another resource that would not deliver what was promised:

*“you know, it just all, it just always feels like, digital solutions are lauded as something that’s really, that’s gonna, you know, change everything, but they never quite give us what we need and they never quite work where we want them to work (MAN-006)*.

However, across our sample of practitioners, there remained enthusiasm for the development of a DHI for these CYP and how well-suited this would be for young, active technology users. CYP's active use of apps was seen as an easy route in to introducing material that was relevant to their experiences of OCSA and lots of suggestions were made about making the DHI as inviting and accessible as possible and making sure that visual material such as videos and images should be used as well as textual content:

*“Erm, I mean certainly having videos as part of your app, where there are, erm, interactions between people, conversations between people, perhaps, you know, it might be a situation, er, someone who’s, who is being groomed, erm, young people could watch those and, and comment on them.. you can do a much higher quality… role-play within, within an app as a video” (MAN-001)*.

Of interest were comments about allowing CYP to be able to make choices about how they would like to use the DHI rather than being too prescriptive and offer the opportunity to personalize the content and to share stories. This included the opportunity to manage the time spent using the DHI across all the material and being interactive with the content and possibly other users. There were also cautions about the amount of text that CYP would be exposed to impacting negatively on their willingness to use the DHI.

#### Subtheme 3: staff need training and support

Despite discussion of every new DHI creating more work for practitioners, when specifically discussing the development of a DHI for OCSA there was reference made to what support may be offered to practitioners and whether they would be involved in the use and monitoring of the intervention:

*“I definitely think there needs to be some really good training, in terms of what the modules that are offered are, erm, and how you as their, as their professional would find out what modules they’ve selected, so how, how you would know that, and how you would know what this young person’s doing” (MAN-010)*.

Practitioners identified a need for training to be at the heart of the DHI development while at the same time expressing concerns about the additional demands that this may make on practitioners. This resulted in suggestions that using a training video, or having the DHI available to them to explore, might be preferable to formal training. A smaller number of practitioners felt that training may not be expected by staff or required. Outside of training it was felt that support would be needed in engaging practitioners to get involved in the use of a DHI for OCSA, with specific suggestions about how this might be achieved. This included the use of posters next to the staff coffee machine, having the DHI on the weekly meeting agenda, and engaging with staff “early adopters” as being agents for change.

*“I think, probably you, it’d probably be about getting someone on the inside to help, to bring things to team meetings, for it to be put on the agenda every week, you know, erm, then, people will start thinking about it and knowing about it” (ED-010)*.

It was also evident that while practitioners could draw on their experiences of using DHI with their client group, this did not relate to working with CYP who had experienced OCSA. This raised concerns about the practicalities for practitioners of introducing the DHI to their client group when questions about online abuse were rarely asked, there was limited understanding about the problem or the impact on CYP, and there was no-one to turn to if questions were asked about the DHI that they could not address. It was felt that practitioners would be able to surmount these problems if they received some encouragement within the service and knew the content of the DHI so that they would be able to discuss this with the young person in a child sensitive way. It was also felt that it was important to know who was there to support practitioners with the use of the DHI:

*“… just, knowing how to contact you probably, you know if that was needed, if, the team felt the want, just to have sort of contact information, and, availability when you can, if needed, do you know, that, I think that would be enough probably” (ED-007)*.

However, underneath the expressed need for training and support, there were inevitable concerns about the additional resources needed for practitioners if they wished to use this DHI with their clients in a meaningful way and the implications that this may have on the service and their other clinical work.

## Discussion

This qualitative study sought to address practitioners' views of how DHIs may play a role in OCSA service delivery and to our knowledge is the first study to do so. This issue seemed particularly pertinent to address as OCSA impacts a substantial number of CYP (63) and there is a growing awareness of this among CAMHS providers, but there appear to be few resources for mental health practitioners to draw on (28). Current access to CAMHS is universally poor (64) and there is growing evidence post-COVID-19 of the need for sustained implementation of digital tools and interventions to encourage both help-seeking and competence by CYP (65, 66). There is also evidence that DHIs could cater to diverse mental health problems at scale for CYP (67, 68). Within the current study, our results suggested that DHIs that responded to OCSA were of particular interest to practitioners given the perceived lack of resources available to them and were seen as potentially stand-alone interventions or ones which would complement existing therapy. It was also suggested that a DHI may reduce waiting-list times and, potentially, positively impact the need for in-person interventions, also noted across other studies (45). Support for DHIs was framed in the context of a “preferred medium” for CYP as it offered a space where they may experience less embarrassment when learning ways of managing their distress and, in addition, equip them with better skills to manage technology in a safer way. As noted by Bell (52) smartphone ownership or private Internet access among CYP within CAMHS is increasingly universal, although, in their study, clinicians were significantly more interested in using technology for mental health support than the young service users who participated in the study. Rather than having digital devices removed or limiting time online as a way of managing further OCSA, there was a suggestion from practitioners that CYP may benefit from what online platforms may bring, learn new ways of staying safe, alongside being able to use a medium that does not mark them out as different from other people. It has been noted in related studies that many CYP value the anonymity associated with DHIs which affords a sense of confidentiality that often seems absent from more traditional in-person interventions (69). Practitioners also felt that CYP might find the DHI empowering in that they could choose how they used it and may also elect to share this with important others, such as carers. Research by Goh et al. (70) found that digital technologies enlarge the space for adolescents to access health services information on their own terms, and provide anonymity, which leads to a sense of safety if access is not curbed by gatekeepers. Other studies have identified that if CYP can access information which is tailored to their needs, they can make informed decisions, which will contribute to improved health outcomes because their better understanding expands their sense of individual agency (71).

There were however challenges identified by practitioners using DHIs to support CYP exposed to OCSA. The first of these related to concerns about who the DHI was suitable for given that technology had played a part in all CYPs' abusive experiences. Suitability was addressed in a systematic review of barriers and facilitators to engagement with DCIs (45). However, suitability was defined as “the degree to which the DHI is in line with daily activities” rather than in relation to extant vulnerabilities. Their results suggested that a proportion of CYP decided not to participate in interventions because they were busy and could not integrate DHIs into their everyday life. They did, however, like time flexibility and the fact that using a DHI meant that they could bypass long waiting lists. Other studies do not make reference to the suitability of using DCIs in related areas such as healthy romantic relationships (35) or cyberbullying (72) nor is there any explicit reference to this in DCIs which focus on forms of OCSA (41, 43). Related to suitability were concerns by practitioners about encouraging CYP to spend more time looking at screens (when using a DHI) as opposed to engaging in in-person relationships. This is a very topical concern given that media use during adolescence can undermine the development of prosocial behavior (73). However, a recent nationally representative study of US adolescents showed self-reports of greater empathic concern and perspective-taking when using social media for connectedness (74).

The greatest concern for practitioners was the absence of a therapeutic relationship when using DCIs and how this may be particularly important in relation to OCSA which will have undermined the ability of CYP to trust others. This concern has been expressed in other studies (75) although a recent scoping review indicated that in youth populations, more evidence is needed to determine the relationship between DCIs and the therapeutic alliance (76). This concern about the lack of an in-person therapist was also linked to the management of the content in a DHI related to OCSA. Others have noted the need for transparency about what CYP may be exposed to and the difficulties in monitoring how DHIs may be used (45). Practitioners felt that explicit content may trigger distressing and painful memories which, in the absence of a practitioner, may be difficult to contain. Other DHI research with suicidal adolescents has also noted the need to consider vulnerabilities related to specific mental health presentations to avoid potential trauma-related triggers (71). Other safety concerns which may be unique to CYP exposed to OCSA were anxieties as to whether the use of the ICT may increase the risk of exposure to a perpetrator or the exchange of sexual media with peers. Too often these anxieties, particularly by people involved in child protection, result in the removal of smartphones from CYP. This is associated with CYP reporting that when OCSA takes place they would not seek help for fear of punishment such as having their phones monitored or removed (77, 78). One final concern related to digitally marginalized youth and those who struggle with technology and lack a private space in which to access DHIs. However, while the evidence base is still limited there is an emerging body of research which indicates that DHIs are a promising option to meet the mental health needs of socioeconomically and digitally marginalized CYP (79) although more high-quality research is needed to bridge the digital divide. Practitioners also expressed concern about the personal toll that promoting a DHI or using it with existing clients would result in more work which may be seen as threatening to people who saw themselves as insufficiently knowledgeable about technology and about OCSA.

Practitioners were asked to consider what they felt was required when developing DHIs for CYP exposed to OCSA. For some practitioners, there were fears that a DHI might be seen as “fobbing off” a young person who had been on a referral waiting list and that there may be an unwillingness to refer clients to the DHI because experience had shown that DHIs never quite worked in the way that they were supposed to and quickly became outdated. This latter concern is particularly pertinent for DHIs related to OCSA and considers issues related to the rapidly changing digital environment that may not only impact how we can reach the targeted population but may impact the content and the resources offered on the DHI (80). However, overall, practitioners showed enthusiasm about a DHI that would be available for CYP exposed to OCSA which is evidenced across a number of other studies (52, 75). A need was expressed to make the DHI as inviting and accessible as possible and to offer CYP choices about how they could use it. A systematic review of children's engagement identified that CYP prefer DHIs with features such as videos, limited text, personalization, text reminders and the capacity to connect with others (45). While there were issues about DHIs creating more work and needing more resources there was also interest in further training about working with the DHI and support for staff and suggestions about raising awareness about its availability which has been noted in other studies (51). Concerns about the content of the DHI were also revisited along with practitioners wanting the ability to monitor progress in how the DCI was being used and offer support as necessary. This also raised issues around data privacy and whether a DHI was a “safe space”. Data security is clearly a key consideration for DCIs targeting CYP, especially in relation to sensitive content such as personal diaries stored on a mobile device (81). An earlier systematic and meta-review (82) concluded that developers should consider CYP's opinions, concerns and requirements concerning data security and privacy of DHIs, and accounts of young users about the development of an online mental health clinic indicated that data-security was central and potentially impacted maintaining trust and privacy (83).

The importance of end users (both practitioners and CYP) in the design and development of DHIs to ensure they are engaging, feasible and effective is a recurrent theme across studies (45, 84, 85). In the context of CAMHS it has been noted that despite practitioners holding positive attitudes towards using technology in patient care this largely involved helplines and websites. Within this study, newer technologies were rarely used by practitioners, and it was felt that a lack of knowledge and concerns about safety and reliability may account for slow uptake in these services (53). Our current study would suggest that practitioners are uncertain about working with CYP who have been exposed to OCSA and are receptive to the idea of a DHI which may reduce waiting lists and offer an alternative or complimentary intervention. However, like the findings from other studies issues such as lack of time to invest in their use or further training, anxieties about content suitability and safety along with data security concerns, anxieties about an unsupported DHI, and lack of evidence about its effectiveness may still act as barriers to its uptake within services.

OCSA is a growing problem, much of it perpetrated by peers and involving the misuse of sexual images and resulting in psychological and reputational harm to many CYP. Our results indicate a need for staff training in children's services as a core requirement to enable the identification and management of OCSA as well as skills to reduce future harm. While there is a growing awareness of technology-facilitated harms for CYP, there are insufficient resources to meet existing needs. DHIs when used in healthcare services may prove a useful resource for practitioners meeting multiple demands in terms of upskilling staff as well as providing CYP as service users with information sourced when needed and in privacy. More research is needed on OCSA DHI app development to ensure user engagement and to overcome concerns by practitioners about safety in the absence of a supportive therapeutic relationship. Recent research has been able to demonstrate the utility of “relational agents” (such as chatbots) to engage and respond to users and to potentially help reduce provider burden (86) and this may be a way to address some of the concerns expressed in the current study.

### Limitations

The current study used a qualitative design with a reflexive thematic analytical approach to analysis. A sample of 25 participants in such a study is in keeping with this method and while we were aware of a tool for determining sample sizes in thematic analysis research, instead we chose to follow the argument presented by Braun and Clarke. The focus was on understanding the experiences of professionals working with CYP who have experienced OCSA. There were more women than men in our sample and there was low ethnic diversity. Certainly, in Edinburgh, these demographics are likely to reflect both service providers and service users whereas in Manchester this may not be the case. Both issues need addressing in future research as both cultural diversity and sex and gender may impact on how comfortable and appropriate practitioners may feel in relation to sexual content included in a DHI app for CYP subjected to OCSA. The sample was recruited from two sites, and we do not know whether this influenced the types of cases that participants were exposed to, although it is likely in relation to CAMHS services this was the case. A larger sample size across more sites may have allowed us to examine this in our analysis.

## Data Availability

The datasets for this article are not publicly available due to concerns regarding participant/patient anonymity. Requests to access the datasets should be directed to the corresponding author.
