# Supplementary material for: What are practitioners' views of how digital health interventions may play a role in online child sexual abuse service delivery?
Source: Front Digit Health. 2024 Mar 20;6:1325385. doi: 10.3389/fdgth.2024.1325385 (PMC10987754; doi:10.3389/fdgth.2024.1325385)
Supplement: Supplementary file 1 [file Datasheet1.pdf]

Study Title: A qualitative study of professional stakeholders' perceptions about the uptake of a digitally mediated intervention for young people who have experienced online sexual abuse and its integration into existing NHS and e-therapy infrastructure.

Topic guide A for interviews or focus groups.

General Interview Guide

Equipment required:

- Participant Information Sheet (in case participant wants to see sheet again)
- Consent form (in case participant wants to see consent form again)
- Video recording (if interview via video conference) or phone recording equipment (if interview via phone)

Prior to interview:

- Participant Information Sheet – any questions?
- Consent form – explain video/audio recording and limits of confidentiality.
- Demographic data questionnaire (separate sheet)
- Technology experiences questionnaire (separate sheet)

Welcome and introduction to interview

Introduction:

Introduce self, welcome, and thank participant for attending the interview. Ensure that the participant is comfortable. Ensure technology is working.

Consent:

Re-confirm informed consent is still valid, and participant still wishes to take part.

Interview details:

Outline interview procedures and expected length of time for the interview.

Remind the participant that the interview will be video/audio-recorded, that participation is voluntary, that they have the right to withdraw at any point without penalty and offer pauses and breaks.

Confidentiality:
Explain confidentiality – everything the participant says during the interview will remain confidential; however, if the participant discloses anything which suggests risk to self or others, the researcher will need to inform the named healthcare contact.

Explain the purpose of the study and take any questions:
"Thanks again for meeting with me today. You are being invited to take part in a research study that aims to understand healthcare professionals' perspectives about what will facilitate the uptake of a digitally-mediated intervention designed to support young people who have experienced online sexual abuse, and its future integration into existing services provided by the NHS and e-therapy providers. OSA involves being asked or persuaded to take part in sexual activities, whether someone is aware or not of what is happening (for example, being coerced into sharing sexual images of oneself, taking part in sexual activities via a webcam or smartphone, having sexual conversations by text, or being groomed, abused and exploited which may then lead to contact abuse) via any device connected to the Internet and across multiple platforms and applications where technology is involved at any stage. During the interview, we will talk about your experiences, views and ideas about working with YP-OSA. In the interview, 'digitally-mediated intervention' refers to any digital tools that can be used to support mental healthcare, including apps, computer programmes, wearables etc. The interview will take approximately 60 minutes to complete. With your permission, the interview will be audio-recorded and then transcribed and anonymised so that we have a record of what has been said. Sometimes we may wish to use quotes that you provide during the interview in publications, but we would always make sure that you would not be identifiable. Do you have any questions at all?"

Seek consent to continue and to audio-record the interview (if applicable).

Inform participant that no personal identifiable data will be recorded and a participant number will be allocated to them.

Topic	Questions
1. **Staff's own experiences with online interventions. (Probe a bit, but if none, move on to question 3).**	What experiences have you had in delivering online interventions (outside of replacement f2f meetings)?<br><br>***Probe:*** *Tell me about the last time…*<br>***Probe:*** *What worked?*<br>***Probe:*** *What didn't work?*
2. **Experience of adapting face-to-face interventions to an online environment. (Probe a bit, but if none, move on to question 3).**	Have you been involved in the adaptation of face-to-face interventions to an online environment?<br><br>***Probe:*** *Tell me how you have gone about doing this in the past?*<br>***Probe:*** *What happened?*
3. **Understanding of YP-OCSA**	What do you consider YP-OCSA to include?<br><br>***Probe:*** *Would you routinely ask about this during your assessment?*<br>***Probe:*** *How might you explore this?*
4. **Current service approaches to YP-OCSA**	What is currently offered in your service to YP-OCSA?<br><br>***Probe:*** *Tell us any support / intervention / assessment that you know of.*<br>***Probe:*** *Which of these are ones that you have used? (probe why they have used these options / probe why they have not used any support/intervention/assessment).*
5. **Specific interventions used with YP-OCSA**	Think about a young person that you worked with who had experienced OCSA.<br><br>***Probe:*** *What overall approach did you use? Why did you use this approach?*<br>***Probe:*** *What specific therapeutic goals did you have? How were these goals derived? (did the clinician or the YP set the goals, etc)*

**6. Specific issues encountered**	What specific issues did you encounter when working with this, or other, young people who have experienced OCSA?<br><br>***Probe:*** *How did you manage these therapeutically?*<br>***Probe:*** *What might you have done differently? Would you do the same again?*
**7. Experiences of mentalization approaches (Probe a bit, but if none, move on to question 3).**	What are your experiences of using a mentalization approach with young people?<br><br>***Probe:*** *If some experience, what are your views on how such an approach might be used in an online intervention for young people exposed to OCSA?*
**8. Expectations about an intervention**	What are your expectations of what an online intervention might target for young people with OCSA?<br><br>***Probe:*** *Think about this in relation to a YP-OCSA you have worked with. What would help that YP? Is there anything you avoid? Why?*
**9. Who might benefit from an online intervention**	Which YP might benefit from an online intervention?<br><br>***Probe:*** *Tell us why you think this?*<br>***Probe:*** *Which young people might not benefit? Why?*
**10. Advantages of online interventions**	What do you think are the advantages of an online intervention being offered / delivered to YP-OSA?<br><br>***Probe:*** *For the young person*<br>***Probe:*** *For professionals*<br>***Probe:*** *For the service*
**11. Disadvantages of online interventions**	What do you think are the disadvantages of an online intervention for YP-OSA?<br><br>***Probe:*** For the young person?<br>***Probe:*** For professionals?<br>***Probe:*** For the service?
**12. Embedding into routine services**	How might a digital resource such as i-Minds (an online mentalisation based

	intervention) be used as part of your routine service?<br>***Probe:*** *Where do you think it would best fit?*<br>***Probe:*** *What would be the barriers to this? Is there anything that would get in the way of you using or directing a YP to this online intervention?.*<br>***Probe***: *What impact (if any) would this have on your workload/practice?*
**13. Referring into iMinds**	What do you think might impact referrals into iMinds?<br><br>***Probe:*** *For the young person?*<br>***Probe:*** *For professionals?*<br>***Probe:*** *For the service?*
**14. Uptake/barriers to uptake of iMinds**	What might facilitate the uptake of iMinds within your organisation?<br><br>***Probe:*** *For the young person?*<br>***Probe:*** *For professionals?*<br>***Probe:*** *For the service?*<br><br>What might get in the way / act as a barrier to the uptake of i-Minds within your organisation?<br><br>***Probe:*** *For the young person?*<br>***Probe:*** *For professionals?*<br>***Probe:*** *For the service?*
**15. Continuing engagement with iMinds**	What do you foresee would facilitate or act as barriers to continued engagement in the intervention?<br><br>***Probe:*** *What would help?*<br><br>***Probe:*** *What would make it more difficult / harder for young people to remain engaged?*<br><br>What might get in the way / act as a barrier to continuing engagement with i-Minds within your organisation?<br><br>***Probe:*** *For the young person?*<br>***Probe:*** *For professionals?*

	Probe: *For the service?*
**16. Support for staff**	What support do you think staff would need?<br><br>***Probe:*** *Training?*<br>***Probe:*** *Extra time?*<br>***Probe:*** *Ongoing support from the project team?*
**17. Scaling up of iMinds**	What are your thoughts whether the intervention could be scaled up and integrated within multiple services?<br><br>***Probe:*** *How might this happen?*<br>***Probe:*** *What might get in the way?*

Interview closedown

"Is there anything else that you would like to tell me that we haven't discussed, but you think might be relevant when thinking about uptake of a digital intervention for YP-OCSA?"

"How have you found this interview today?"

"How do you think this interview could be improved for future participants?"

"Ok, I'll now switch off the video/audio recorder."

End of interview

Thank the participant for taking part.

Explain what will happen with the information provided.

Check that the participant is still happy for you to use all the information provided.

Ask the participant whether they would like to receive a summary of the results.

Ask the participant whether they have any questions.

Offer a support phone call: "sometimes people take part in an interview and afterwards they have more questions to ask, or they have been worrying about something they said. If you like, I can call you tomorrow just to check if any of this is happening for you – would you like me to do that?"

i-Minds: A digital intervention to improve mental health and interpersonal resilience in young people who have experienced online sexual abuse: a non-randomised feasibility study with a mixed-methods design

Workstream 1 – Digital translation of the mentalisation-based intervention and digital platform development
